# Detailed Analysis of Protein Topology of Extracellular Vesicles–Evidence of Unconventional Membrane Protein Orientation

**DOI:** 10.1038/srep36338

**Published:** 2016-11-08

**Authors:** Aleksander Cvjetkovic, Su Chul Jang, Barbora Konečná, Johanna L. Höög, Carina Sihlbom, Cecilia Lässer, Jan Lötvall

**Affiliations:** 1 Krefting Research Center, Institute of Medicine, University of Gothenburg, Gothenburg, Sweden; 2 Institute of Molecular Biomedicine, Faculty of Medicine, Comenius University, Bratislava, Slovakia; 3 Department of Chemistry and Molecular Biology, Faculty of Natural Sciences, University of Gothenburg, Gothenburg, Sweden; 4 Proteomics Core Facility, University of Gothenburg, Gothenburg, Sweden

## Abstract

Extracellular vesicles (EVs) are important mediators of intercellular communication that change the recipient cell by shuttling lipids, RNA, or protein cargo between cells. Here, we investigate the topology of the protein cargo found in EVs, as this topology can fundamentally influence the biological effects of EVs. A multiple proteomics approach, combining proteinase treatment and biotin tagging, shows that many proteins of cytosolic origin are localized on the surface of EVs. A detailed analysis of the EV proteome at the peptide level revealed that a number of EV membrane proteins are present in a topologically reversed orientation compared to what is annotated. Two examples of such proteins, SCAMP3 and STX4, were confirmed to have a reversed topology. This reversed typology was determined using flow cytometry and fluorescent microscopy with antibodies directed toward their cytoplasmic epitopes. These results describe a novel workflow to define the EV proteome and the orientation of each protein, including membrane protein topology. These data are fundamentally important to understanding the EV proteome and required to fully explain EV biogenesis as well as biological function in recipient cells.

Intercellular communication is essential for multicellular organisms to maintain homeostasis and can be mediated by direct contact or through secretion of molecules such as bioactive proteins and lipids. In addition, most cell types, including immune cells and cancer cells, secrete extracellular vesicles (EVs) that can influence recipient cell phenotype^1^,^2^. EVs are lipid bilayered membrane vesicles with a diameter of 30 ~ 1000 nm carrying multiple biologically active cargo components such as proteins, nucleic acids (mRNAs, miRNAs, and small RNAs), and lipids. Through the transfer of its cargo, EVs mediate diverse biological functions that include immunomodulation^3^, cancer progression^4^, and epigenetic reprogramming^5^. In addition, since EVs have been shown to contain disease specific markers, their potential as diagnostic markers have generated great attention^6^,^7^.

Cells secrete different types of EVs, often divided into exosomes and microvesicles. Exosomes are released by fusion of the multivesicular body with the plasma membrane, and microvesicles bud out from the plasma membrane directly^8^. Although there are some differences in the size and composition of these EVs, it remains impossible to completely separate exosomes and microvesicles with the currently available purification methods. Therefore, we use the term “EVs” throughout this publication.

In-depth large-scale proteome analysis can contribute to the understanding of the biogenesis and functional role of EVs as well as to the discovery of diagnostic markers. EVs purified from various cell culture media^9^ and body fluids–e.g., urine^10^, blood^11^, saliva^12^, and breast milk^13^–have been analyzed with proteomics technology. Those proteome data are well-organized in the EV databases EVpedia^14^,^15^ and Vesiclepedia^16^. Many EV surface proteins are transmembrane proteins^14^, but those that are not may be non-covalently bound to the EVs and could be travelling with the EVs between cells. As more functional studies of EVs are published, it becomes increasingly essential to determine the detailed orientation of proteins on or inside of EVs. From a clinical perspective, understanding the details of the surface proteome of EVs is essential for developing EVs as biomarkers for disease. Importantly, very little is known about the topology of different proteins identified in different EV samples such as biofluids, tissues, or cell supernatants.

Here, we present a novel work-flow designed to identify the proteins that are localized on the surface of EVs in EV isolates. To identify the surface proteome, we designed a multiple-approach proteomics study combining proteinase treatment and biotin tagging. Using a proteinase and a biotinylation reagent that are both impermeable to a lipid bilayer^17^,^18^, we assessed the surface proteome of the EVs for digestion or labeling. The EVs were isolated from the HMC-1 mast cell line through differential ultracentrifugation followed by density gradient floatation. The isolated EVs were treated with either proteinase K (PK) to completely digest the surface proteins or with trypsin/Lys-C and subsequently biotinylated. Through the combination of methods described here and subsequent label-free comparative and quantitative proteomics, we were able to describe an EV proteome where the luminal contents of EVs can be distinguished from the proteins present on the surface of the vesicles. Additionally, through a detailed evaluation at the peptide level, we clarified the topology of many transmembrane and lipid-anchored proteins in the EVs and identified which of these proteins exhibited unconventional “inside-out” topology.

## Results

### Overall strategy for identifying surface-accessible proteome of EV isolates

**Figure 1:**
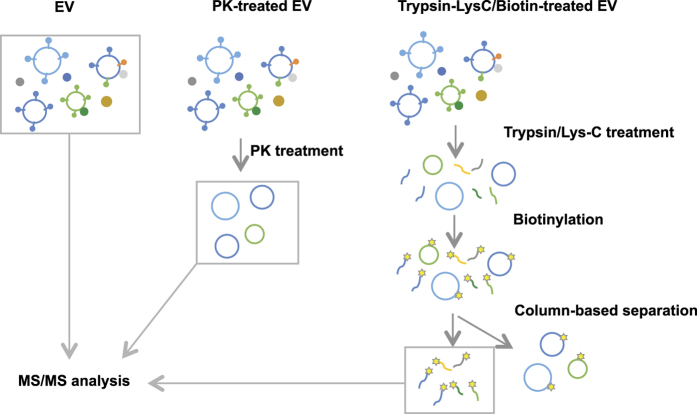
Schematic illustration of study. EVs from HMC-1 cells were treated with proteinase K (PK) or trypsin/Lys-C. Trypsin/Lys-C treated EVs were further treated with sulfo-LC-biotin, and biotinylated peptides were isolated by column based separation. EVs, PK-treated EVs, and biotinylated peptides were analyzed with LC-MS/MS.

To define the luminal and the surface-accessible proteome of EVs, we designed a multiple-approach proteomics study as described in Fig. 1. For the first approach, EVs were treated with PK to remove surface-accessible proteins. Since PK cannot penetrate through the lipid bilayer, it is most likely that proteins susceptible to PK degradation are localized on the surface of EVs. For the second approach, EVs were treated with trypsin and Lys-C to digest surface-accessible proteins and then biotin tagged. Biotinylated peptides were isolated using column base separation. Finally, non-treated, PK-treated, and biotinylated samples were analyzed with liquid chromatography-tandem mass spectrometry (LC-MS/MS), and bioinformatics were performed to compare data of the different samples.

### Isolation and characterization of EVs from HMC-1 cells

**Figure 2:**
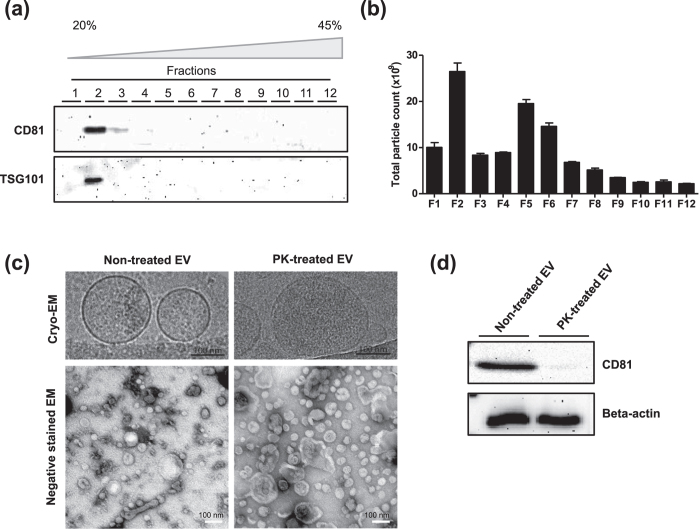
Characterization of EVs and PK-treated EVs. (**a**) Western blot analysis with EV markers, CD81 and TSG101, in 12 fractions from OptiPrep density gradient. (**b**) The particle number in each OptiPrep fraction was analyzed by nanoparticle tracking analysis. (**c**) Cryo-EM images of non-treated and PK-treated EVs. (**d**) Western blot analysis of non-treated and PK-treated EVs with CD81 and beta-actin.

As a model system, we isolated EVs from human mast cell line HMC-1 cells. Cells were grown in media complemented with 10% EV-depleted Fetal Bovine Serum for three days. In this culture condition, cell viability was higher than 98% (data not shown). Conditioned media was collected and EVs were isolated through serial centrifugation and ultracentrifugation and further purified by floatation on a density gradient (Iodixanol). A total of twelve fractions were collected and subjected to Western blot analysis to identify vesicular proteins. The classic marker proteins CD81 and TSG101^19^ were detected in fractions 2 and 3 and fraction 2, respectively (Fig. 2a). The particle number of each fraction, which was counted using nanoparticle tracking analysis, showed that most particles were detected in fraction 2 (Fig. 2b). Many particles were also detected in the denser fractions 5 and 6, but did not express the classic surface proteins (i.e., CD81 and TSG101), suggesting that these particles may consist of protein aggregates or different sub-populations of EVs not carrying these classic markers. We collected fractions 2 and 3, diluted them with PBS, and then pelleted these by ultracentrifugation. Finally, EVs were re-suspended in PBS. Cryo- and negative stained-EM micrographs showed that isolated EVs are mostly spherical, vary in diameter, and include a lipid bilayer (Fig. 2c, left panel). Importantly, to avoid the possibility of the EV vesicular structure being compromised by freezing and thawing, all experiments were performed in one step during which the EVs were not frozen at any time.

### Proteomic analysis for defining the EV cargo proteome and the surface-accessible EV proteome

To digest the surface-accessible proteins, EVs were exposed to PK. This enzyme digested the plasma transmembrane protein CD81, but did not digest the luminal protein beta-actin (Fig. 2d). Cryo- and negative stained-EM micrographs revealed that PK treatment did not change the integrity or morphology of EVs (Fig. 2c, right panel). Taken together, these results suggest that PK efficiently digests proteins localized on the surface of the EVs without compromising the integrity of the EVs themselves.

**Figure 3:**
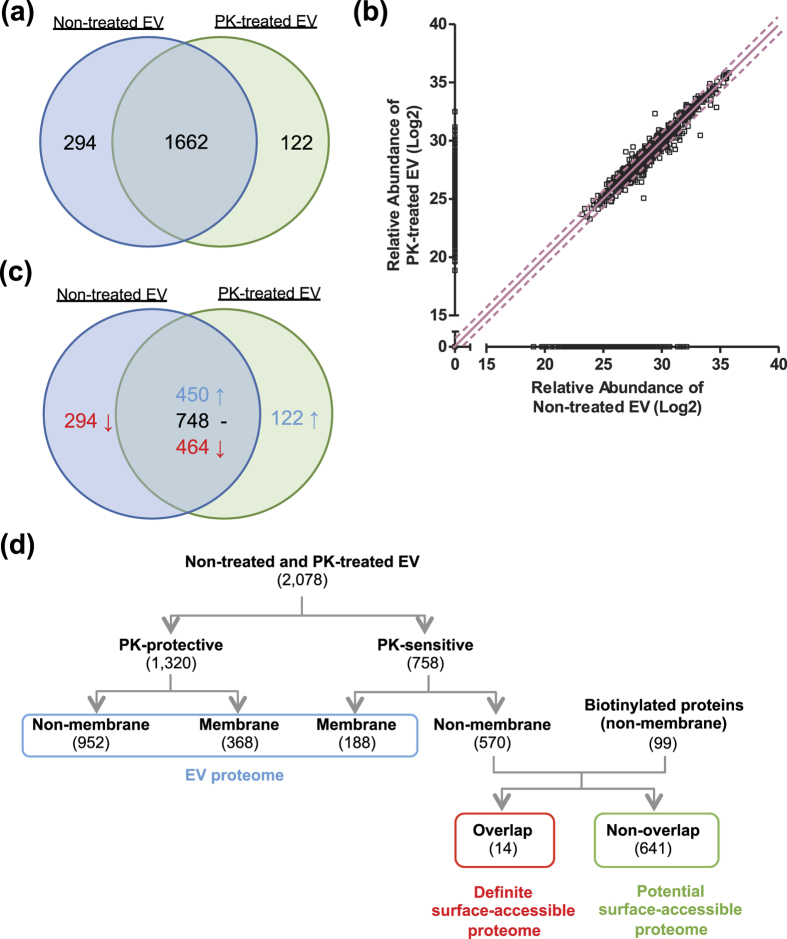
Defining EV and surface-accessible proteome. (**a**) Venn diagram of non-treated and PK-treated EVs proteome. The number present in the circle represent the total number of identified proteins in particular data set. (**b**) Plot of log2 value of relative abundance of proteins from non-treated and PK-treated EVs. Line and dotted line indicate half and 2-fold change, respectively. (**c**) Among the common proteins, proteins are divided into three groups–2-fold increase, 2-fold decrease, and no change after PK treatment–based on relative protein abundance. (**d**) Hierarchical diagram of defining EV and surface-accessible proteome.

Non-treated and PK-treated EVs were lysed and column associated trypsin digestion was conducted with filter-aided sample preparation. Finally, the eluted tryptic peptides were subjected to LC-MS/MS. From the proteomics analysis, we identified 1956 and 1784 proteins from non-treated and PK-treated EVs, respectively. As shown in the Venn diagram (Fig. 3a), 1662 proteins were identified in both EV preparations, whereas 294 and 122 proteins were uniquely identified in non-treated and PK-treated EVs, respectively. The relative abundance of different proteins was obtained using the MaxQuant software and was plotted (Fig. 3b). Based on the relative protein abundance, proteins were divided into two groups–“PK sensitive” and “PK protected”. Among the 1662 common proteins, 748 proteins did not change markedly in abundance, whereas 450 and 464 proteins were relatively increased and decreased after PK treatment, respectively (Fig. 3c). The proteins unique to the non-treated EVs and those that were 2-fold decreased in the PK-treated EVs were categorized as “PK-sensitive” and the rest were categorized as “PK-protected” (Fig. 3d). Next, a sub-division into membrane and non-membrane proteins was conducted based on their primary subcellular localization, according to the Uniprot database (only primary localizations were used). Among 1320 PK-protected proteins, 368 and 952 proteins had membrane and non-membrane localizations, respectively. In addition, among the 758 PK-sensitive proteins, 188 and 570 proteins had membrane and non-membrane localizations, respectively.

In parallel, we also applied an additional method to identify surface-accessible proteins. First, EVs were treated with trypsin and Lys-C, and then biotinylated with sulfo-LC-biotin, which is impermeable to the lipid bilayer. The biotinylation reagent labels free amine groups of lysine residues and the N-terminal of amino acids, both of which are theoretically made more abundant through treatment with trypsin and Lys-C. Digested and biotinylated peptides were isolated by column based separation and analyzed with LC-MS/MS. In total, 155 proteins were identified with at least one biotinylated peptide. Similarly, as described above, proteins were divided into membrane (56 proteins) and non-membrane (99 proteins) based on their primary localization (Fig. 3d).

Many membrane proteins are exposed on the surface of EVs, allowing them to be digested by proteinases as well as labeled with biotin. As these proteins are embedded in the membrane, we considered these to be part of the EV proteome even though they potentially are susceptible to the aforementioned reagents. Taking this into account and applying a strict analytical approach, we only selected non-membrane proteins from the PK-sensitive and biotinylated proteins and considered these to definitely contribute to the surface-accessible proteome. By comparing non-membrane proteins from PK-sensitive and biotinylated proteins, we defined 14 overlapping proteins that definitely contribute to the surface-accessible proteome and 641 non-overlapping proteins as potentially contributing to the surface-accessible proteome. Overall, 655 proteins are considered to contribute to the surface-accessible proteome. In addition, non-PK-degraded proteins (1320 proteins) and membrane proteins from PK-sensitive proteins (188 proteins) are considered to contribute to EV proteome, as membrane proteins most likely are associated with the EV lipid bilayer. A complete list of proteins is provided as Supplementary Table S1. Importantly, classic cytosolic EV marker proteins, including syntenin, TSG101, and proteins in the Annexin and Rab families^19^ (except Rab31), are defined as EV cargo proteomes because they were not biotinylated or degraded by PK (Table S2), further confirming the validity of our topological classification of the EV proteome.

### Subcellular localization and gene ontology analysis

**Figure 4:**
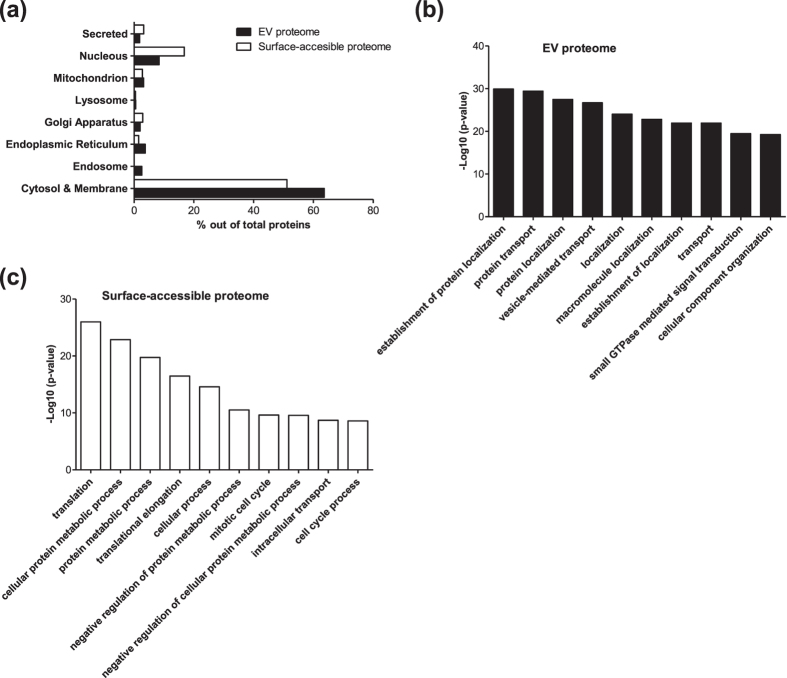
Subcellular localization and gene ontology analysis of EV and surface-accessible proteome. (**a**) Subcellular localization of EV and surface-accessible proteome. (**b**) Top 10 biological process gene ontology terms enriched in EV proteome. (**c**) Top 10 biological process gene ontology terms enriched in surface-accessible proteome.

Next, we analyzed the subcellular localization and biological process GO terms of the EV cargo and surface-accessible proteome. Subcellular localization of the surface-accessible proteins showed that 51.1% and 16.8% of surface-accessible proteome are cytosolic and nuclear proteins, respectively (Fig. 4a). Notably, the percentage of nucleus proteins in the surface-accessible proteome is twice that of the EV proteome (8.3%). In the GO term biological process analysis, the EV cargo and surface-accessible proteome showed distinct features. The EV proteome is enriched with biological processes including establishment of protein localization, protein transport, protein localization, vesicle-mediated transport and localization (Fig. 4b). By contrast, the surface-accessible proteome is enriched with biological processes including translation, cellular protein metabolic process, protein metabolic process, translational elongation and cellular process (Fig. 4c). Taken together, our results suggest that many cytosolic and/or nuclear proteins are co-isolated with the EVs and are actually localized on the exterior part of the EVs.

### Topology of transmembrane and lipid-anchored proteins

**Figure 5:**
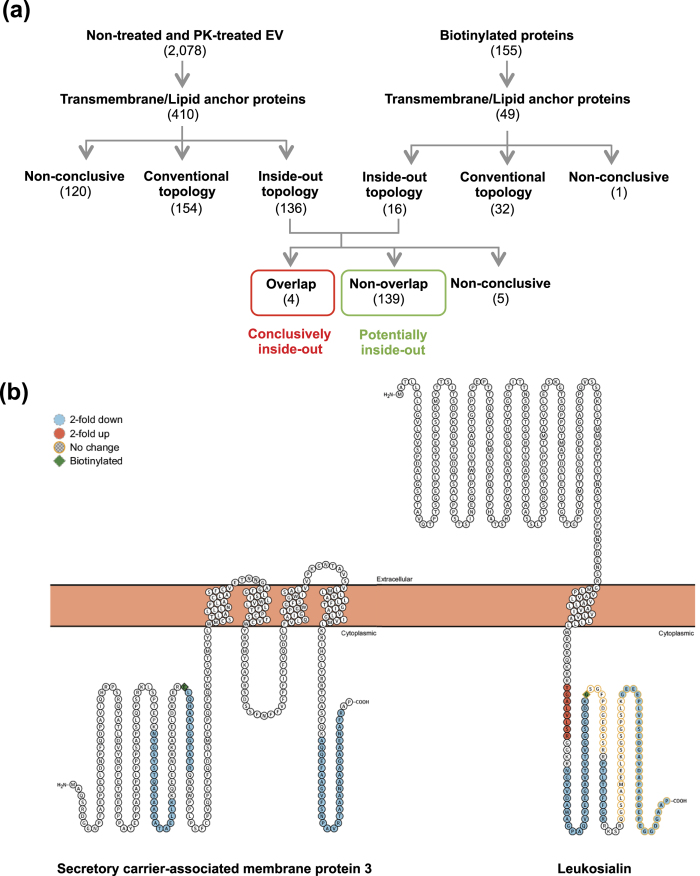
Topology analysis of transmembrane and lipid-anchored proteins. (**a**) Hierarchical diagram of defining topology of transmembrane and lipid-anchored proteins. Proteins were visualized with Protter tool and the information about the identified peptides was integrated. Based on localization of peptides, correct topology and inside-out were defined. (**b**) Topology illustration of two examples of conclusively inside-out proteins. Peptides that were found in LC-MS/MS were visualized by Protter.

Additionally, we analyzed the topology of transmembrane and lipid-anchored proteins in the EVs using the peptide information from the mass spectrometry results. A simple scoring system was used to establish unbiased criteria to determine the topology of membrane proteins. From 2078 proteins of non-treated and PK-treated EVs, 410 transmembrane and lipid-anchored proteins were selected and scored. Topology illustrations of these proteins were obtained using the Protter tool (http://wlab.ethz.ch/protter/, version 1.0, June 2016)^20^ and identified peptides were mapped on topology illustrations. Peptides that are 2-fold enriched in non-treated EV and are localized at the extracellular region were scored as +1. However, if they localized in the cytoplasmic region, they were scored as −1. Likewise, peptides that are 2-fold enriched in PK-treated EVs and localized at cytoplasmic region were scored as +1, but if they localized at extracellular region, they were scored −1. Peptides that had a lower than 2-fold change were not taken into account. Proteins that were scored with a value higher than 0 were considered to have a “conventional topology”, namely their cytoplasmic or extracellular parts are located as annotated by Uniprot. Proteins scored lower than 0 were considered to have an “inside-out topology”. If the score was 0, then no conclusion could be made and they were designated as “non-conclusive”. Among these 410 proteins, 154, 136, and 120 proteins were classified as “conventional topology”, “inside-out topology”, and “non-conclusive”, respectively (Fig. 5a). In the same way, 49 transmembrane and lipid-anchored proteins were selected from 155 biotinylated proteins. The localization of biotinylated peptides of those proteins was also analyzed and similarly scored. If biotinylated peptides belonged to the cytoplasmic region of a protein, that protein was considered to have an “inside-out topology”. Thirty-two proteins were classified as “conventional topology”, sixteen as “inside-out topology”, and one as “non-conclusive” (Fig. 5a). After comparing the inside-out proteins from both the PK and biotinylation lists, we found four overlapping proteins with unconventional topology. We assigned these four proteins as conclusively having inside-out topology and the additional non-overlapping 139 proteins as potentially having inside-out topology. For five proteins, the data were contradictive, so these proteins were assigned as “non-conclusive”. The four conclusively inside-out proteins and total proteins are listed in Supplementary Table S3. Two examples of conclusively inside-out proteins and their identified peptides are shown in Fig. 5b. Clearly, it is conceivable and even likely that some of the potential inside-out proteins *sometimes* are oriented “inside out” and *sometimes* have conventional topology.

### Validation of surface-accessible and inside-out proteome

**Figure 6:**
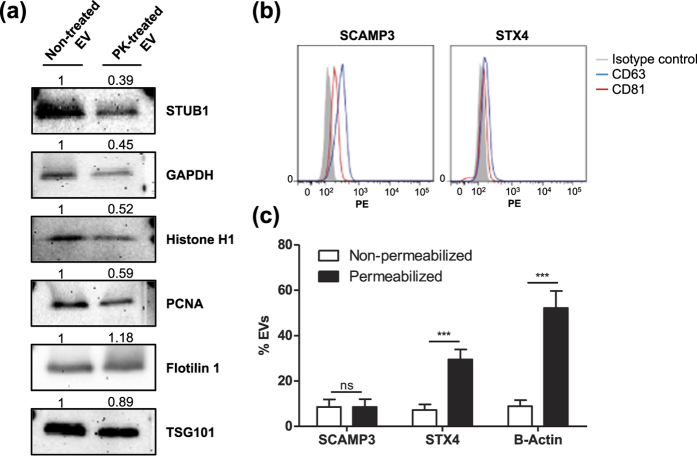
Validation of surface-accessible proteome and inside-out proteins. (**a**) Western blot analysis of surface-accessible and EV proteome. STUB1, GAPDH, Histone H1, and PCNA are surface-accessible proteome, whereas Flotilin 1 and TSG101 are EV proteome. Relative band intensity was measured. (**b**) Flow cytometry of inside-out membrane proteins. EVs were captured by SCAMP3 (left panel) or STX4 (right panel) antibody conjugated beads and then detected with CD63 or CD81. (**c**) The percentage of SCAMP3, STX4, and beta-actin positive EVs were calculated after incubation with or without 0.1% Tween-20.

The findings thus far strongly point to the presence of proteins that are localized on the surface of EVs as well as some membrane bound proteins that are not oriented in their annotated orientation according to Protter. To confirm these findings, we performed a number of validation experiments. Western blots were conducted with both PK-treated and non-treated EVs with probes directed toward the proteins Flotillin-1, TSG101, GAPDH, STUB1, Histone H1, and PCNA (Fig. 6a). Flotillin-1 and TSG101 was used as a marker for the protected luminal part. GAPDH, STUB1, Histone H1, and PCNA are proteins taken from the “definite surface-accessible” group of proteins among which GAPDH and STUB1 are previously annotated to be a component of the cytoplasm, whereas Histone H1 and PCNA are nuclear components. The blot shows that the band intensity of GAPDH, STUB1, Histone H1, and PCNA were diminished in PK-treated EVs compared to those in the non-treated EVs, whereas the TSG101 and Flotillin-1 bands did not change markedly. These results confirm our classification of the surface-accessible proteome by using our multiple-approach proteomics approach, further suggesting that EV isolates contain non-EV proteins that are localized on the surface of EVs.

Flow cytometric analyses were carried out to validate the orientation of the membrane-bound proteins, which our proteomics data suggest contradict current dogma. Magnetic beads were coated with antibodies directed towards the “inside” domain of either SCAMP3 or STX4, proteins taken from the list of “conclusively inside-out” or “potentially inside-out” proteins, respectively. The antibodies are directed toward epitopes that should be present on the luminal/cytoplasmic part of these proteins; therefore, the beads should only bind to inside-out proteins on the EVs. The bead-captured EVs were then probed for the common EV markers CD81 and CD63. A shift in MFI can be observed, suggesting the presence of EVs carrying inside-out proteins as well as CD63 with the correct topology (Fig. 6b). Thus, these flow cytometry experiments show that some EVs indeed harbor proteins with inside-out topology, although this seems to be true for only a portion of the EVs and the EV membrane proteins. To further strengthen this conclusion, we also evaluated these two proteins using wide field microscopy. The EVs were stained using the lipid dye PKH 26 and fluorescently stained with the same antibodies towards the inside domains of SCAMP3 and STX4 with or without permeabilization. Next, we counted the overlapping fluorescence between the PKH dye and the antibodies. This overlap signifies EVs that carry inside-out membrane proteins. Beta-actin was used as a luminal protein control, but only the secondary antibody was used as a control for non-specific binding. Comparing the use of primary antibodies to that of only the secondary clearly shows an increase in co-localization events in all three proteins (Fig. 6c). By permeabilizing the EVs, the substantial increase in co-localization of beta-actin indicates that a certain amount of beta-actin is present on the surface of EVs although the majority of this protein is luminal. This finding also confirms the success of permeabilization of the EVs. Importantly, the number of co-localized points after permeabilization is greatly increased for STX4 but not for SCAMP3, indicating that only a portion of the EVs carrying STX4 have the inside-out topology, while it appears as SCAMP3 only exists in its reversed orientation. Taken together, these results confirm that a portion of membrane-bound proteins in EVs have an inside-out topology.

## Discussion

There is a rapidly increasing interest in the biology and function of EVs as these vesicles have the potential to be diagnostic biomarkers or used as treatment vectors. However, before EVs can fulfill their clinical roles, multiple obstacles need to be overcome. For example, surface proteome and cargo proteome need to be separately identified and the topology of the EV membrane proteins needs to be determined.

Here we describe the EV cargo with a detailed proteomics perspective. A two-way approach was applied where samples were either subjected to proteinase or sulfo-NHS-biotin, which either degrades or biotinylates proteins with epitopes (lysines and N-terminals) exposed to the surface of the EV lipid bi-layer. Importantly, various research groups have already examined the effect of freezing and thawing on EVs. Because these studies suggest that size and protein contents are affected by freezing and thawing^21^,^22^, we chose to analyze the proteome only in fresh samples. By using comparative and quantitative analysis, we defined 14 proteins as conclusively surface-accessible proteins and 641 proteins as potential/likely surface-accessible proteins (Fig. 2d). Our results suggest that multiple cytosolic and nuclear proteins are present on the surface of EV isolates. These proteins could be connected to the EVs by non-covalent binding, and it is unclear whether these proteins are natural EV surface molecules or whether this is a result of *in vitro* culture conditions.

A more detailed analysis of the EV proteome at a peptide level revealed that in some cases cytoplasmic or rather luminal parts of some membrane proteins seemed to be degraded by PK. This finding led us to form the hypothesis that some of these proteins existed in the membrane in a topologically reversed orientation than previously annotated. Two examples of proteins with inside-out topology, SCAMP3 and STX4, were validated using several experimental techniques (Fig. 6). Interestingly, most of the Rab proteins, which are lipid anchored molecules, were classified as having either an inside-out topology or no conclusion (Table S2). We found that Rab5b protein has an inside-out topology and a previous study found that Rab5b is localized at the surface of EVs^23^. In addition to this observation, some studies show that RNA can be localized on the surface of EVs as suggested by the effects of RNase treatment^24^, even though most studies claim that the RNA is protected inside of EVs^1^,^25^,^26^. Our results and previous studies argue for the existence of inside-out EVs or the inside-out topology of some specific EV components, although a more in-depth study is needed to confirm these conclusions.

The presence of “inside-out proteins” is puzzling and the potential significance of this phenomenon even more so. One explanation for this is that the EV originates from an area where protein translocation has taken place, such as the endoplasmic reticulum^27^. Additionally, protein topology is influenced by multiple factors^28^ such as the local lipid composition^29^. It can be speculated that during biogenesis, if an ER vesicle transports a protein during its translation to the late endosome or the buddying MVB, the altered lipid environment would promote a non-conventional topological orientation. Alternatively, the altered environment might be enough to induce an already fully inserted protein to change its conformation.

Recent studies on EV diversity revealed by cryo-EM showed that EVs have a very varied morphology, not only single membrane vesicles but also double membrane vesicles^30^. In such cases, the inner vesicle is protected by the membrane of the outer vesicle, which must be considered when interpreting both the PK treatment and biotinylation data. Another possible reason for proteins having reversed topology could be that the stability of the membrane protein is compromised by PK treatment and thus the native topology becomes energetically unfavorable, resulting in some cytoplasmic regions being expelled into the extra-vesicular environment. Alternatively, PK treatment could destabilize some membrane proteins in such a way that luminal sequences that would otherwise be inaccessible to endogenous proteases become accessible and would thus upon degradation appear as missing in the proteomics. However, both of these explanations are speculative and further study is needed.

**Figure 7:**
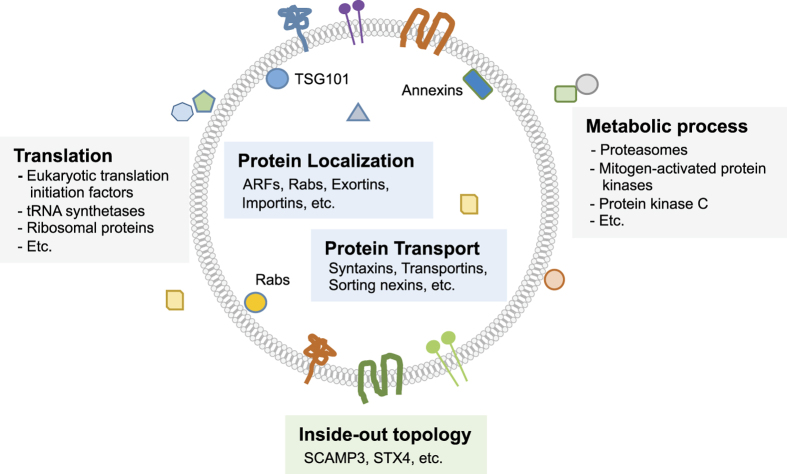
Schematic overview of EV proteome. Selected examples of proteins are illustrated. Proteins in EV isolates can be localized either inside (blue box) or on the surface (gray box) of EVs. In addition, some of transmembrane and lipid-anchored proteins have inside-out topology (green box).

In conclusion, our study provides a global map of the topology of the proteome of EVs isolated from HMC-1 cells (Fig. 7). Many proteins are localized on the surface of the EV lipid bilayer, and some membrane proteins have inside-out topology. These results give a new insight about the EV proteome and help explain the presence of impurities as the result of EV isolation. Our presented work-flow will be very helpful for the EV proteome field and could be applied to other types of EVs to describe their vesicular protein orientation, possibly explaining some EV function.

## Methods

### Cell culture

The human mast cell line HMC-1 (Dr. Joseph Butterfield, Mayo Clinic, Rochester, MN, USA) was cultured in IMDM media (HyClone) supplemented with 10% EV-depleted fetal bovine serum (Sigma Aldrich), 2 mM L-glutamine (HyClone), 100 units/ml Penicillin, 100 μg/ml Streptomycin (HyClone), and 1.2 U/ml 1-Thioglycerol (Sigma Aldrich). The FBS was ultracentrifuged at 118,000 × g_avg_ (Type 45 Ti rotor, k-factor 178.6, Beckman Coulter, CA, USA) for 18 h and filtered through a 0.22 μm filter to deplete it of EVs, as previously described^31^.

### Isolation of extracellular vesicles

HMC-1 cells were seeded in IMDM media with 10% EV-depleted FBS at a 5 × 10^5^ cells/ml concentration and incubated for three days at 37 °C in a humidified atmosphere with 5% CO_2_. Conditioned media was harvested and centrifuged at 300 × g for 10 min to remove cells. The supernatant was then centrifuged at 16,500 × g_avg_ for 20 min to remove apoptotic bodies and larger particles. Lastly, the supernatant was ultracentrifuged at 118,000 × g_avg_ (Type 45 Ti) for 3.5 h and the resulting pellet was resuspended in phosphate buffered saline (PBS). To obtain a higher purity of EVs, an isopycnic centrifugation, using an iodixanol (OptiPrep, Sigma Aldrich, Saint Louis, USA) gradient, was conducted. EVs in PBS (1 ml) were mixed with 60% of iodixanol (3 ml) and laid on the bottom of an ultracentrifuge tube. A discontinuous iodixanol gradient (35, 30, 28, 26, 24, 22, 20%; 1 ml each, but 2 ml for 22%) in 0.25 M sucrose, 10 mM Tris, and 1 mM EDTA was overlaid and finally the tubes were filled to completion with approximately 400 μl of PBS. Samples were ultracentrifuged at 178,000 × g_avg_ (SW 41 Ti, k-factor 143.9, Beckman Coulter) for 16 h. Fractions (1 ml) were collected from the top to the bottom and subjected to Western blot analysis to identify EV markers. Mixture of fraction 2 and 3 were diluted with PBS (up to 94 ml) and ultrancetrifuged at 118,000 × g_avg_ (Type 45 Ti) for 3.5 h. The pelleted EVs were resuspended in PBS. Protein concentration was measured with the BCA protein assay kit (Thermo Fisher Scientific). EVs isolated by ultracentrifugation with density gradient was used for all the experiments in this study. Importantly, all experiments were done in sequence and EVs were never frozen.

### Electron microscopy

For Cryo-EM, freshly isolated EVs, PK-treated and non-treated, were plunge frozen as previously described^30^, using a Vitrobot Mk2 (FEI; Eindhofen, The Netherlands). Images were acquired using the TVIPS EMMENU 3.0 software and a TVIPS TemCam F224 camera on a FEI CM200 microscope operated at 200kV in low dose mode. For negative stain electron microscopy, PK-treated and non-treated vesicles were put on formvar and carbon coated electron microscopy grids (Cu; 200 mesh) for 5 min. Samples were washed (3x using PBS) and then fixed using 2.5% glutaraldehyde in PBS. After two further washes in filtered water, the samples were stained using 2% uranyl acetate for 1.5 min. Images were taken using a SiS Morada CCD-camera (Olympus, Münster, Germany) on a LEO 912AB Omega electron microscope (Carl Zeiss NTS, Jena, Germany) operated at 120kV.

### Western blot analysis

EV proteins were separated by SDS-PAGE and transferred to a polyvinylidene fluoride membrane. The membrane was blocked with 5% non-fat dry milk in Tris-buffered saline with 0.05% of tween 20 (TBST) for 2 h. The membranes were then incubated with the following primary antibodies: anti-CD81 (H-121, Santa Cruz Biotechnology), anti-TSG101 (Abcam), anti-GAPDH (6c5, Santa Cruz Biotechnology), anti-beta-actin (C4, Santa Cruz Biotechnology), anti-STUB1 (Abcam), anti-KIF14 (Abcam). All antibodies were diluted with 0.25% non-fat dry milk in 0.05% TBST at 4 °C overnight. The membrane was washed with 0.05% TBST and then incubated with the secondary antibody for 2 hours. After washing with 0.05% TBST, the immune-reactive bands were visualized using either SuperSignal West Femto Maximum Sensitivity Substrate (Thermo Fisher Scientific) or Amersham ECL Prime Western Blotting Detection Reagent (GE healthcare) with a VersaDoc 4000 MP (Bio-Rad Laboratories). Band intensity was measured using Image J program and normalized by intensity of non-treated EVs.

### Particle measurement

The number of EVs was measured using ZetaView PMX 110 (Particle Metrix). Each fraction from the OptiPrep gradient was diluted with PBS and measured. The chamber temperature was automatically measured and applied to calculation. Data was obtained from triplicate measurements and each individual data was obtained from two stationary layers with five times measurement in each layer. Sensitivity of the camera was 70. Data was analyzed using the ZetaView analysis software version 8.2.30.1 with a minimum size of 5, a maximum size of 1000, and a minimum brightness of 20.

### Proteinase K treatment

EVs were diluted to a concentration of 860 μg/ml worth of protein and incubated with 20 μg/ml Proteinase K (Invitrogen) and 5 mM CaCl_2_ in PBS for 1 h at 37 °C with gentle vortexing every 15 minutes. The proteinase activity was then inhibited by adding 5 mM phenylmethylsulfonyl fluoride for 10 minutes at room temperature.

### Trypsin/Lys-C digestion and biotin labeling

EVs were treated with a mixture of 20 μg/ml trypsin and 10 μg/ml Lys-C for 2 h at 37 °C. Following proteinase treatment, the EVs were incubated with 20 mM EZ-Link Sulfo-NHS-Biotin (Thermo Fischer Scientific) for 30 min at room temperature. Excessive biotin was quenched using 10 mM hydroxylamine. Separation was performed using FASP sample processing and C18 spin columns desalting according to manufacturer’s instructions.

### LC-MS/MS analysis

The peptides were reconstituted with 15 μl of 0.1% formic acid (Sigma Aldrich) in 3% acetonitrile and analyzed on an Orbitrap Fusion Tribrid mass spectrometer interfaced to an Easy-nLC 1000 (Thermo Fisher Scientific). Peptides (2 μl injection volume) were separated using an analytical column (200 × 0.075 mm I.D.) packed in-house with 3 μm Reprosil-Pur C18-AQ particles (Dr. Maisch, Germany). A gradient was run at 200 nl/min; from 5% B-solvent (acetonitrile in 0.2% formic acid) to 80% B, over 90 min in solvent A (0.2% formic acid). Ions were injected into the mass spectrometer under a spray voltage of 1.6 kV in positive ion mode. MS scans was performed at 120 000 resolution, m/z range 400–1500, MS/MS analysis was performed in a data-dependent mode, with top speed cycle of 3s for doubly or multiply charged precursor ions. Ions in each MS scan were selected for fragmentation (MS2) by collision induced dissociation (CID) at 30% and detection in the ion trap and dynamic exclusion within 20 ppm during 30 seconds was used for m/z-values already selected for fragmentation. Each sample was analyzed twice using first the most intense precursors above threshold 10000 and then the least intense precursors in the second injection.

### Identification and quantification of proteins

Peak lists of MS data were generated and peptides/proteins were identified and quantified using the MaxQuant quantification tool with Andromeda search engine (version 1.5.2.8). The search parameters used were as follows: enzyme specificity, trypsin; variable modification for oxidation of methionine (15.995 Da) and fixed modification for carbamidomethylation of cysteine (57.021 Da); two missed cleavages; 20 ppm for precursor ions tolerance and 4.5 ppm for fragment ions tolerance. *Homo sapiens* reference proteome set from Swiss-Prot database (20196 entries), contaminants, and reverse sequences was used for search. For peptide and protein identification, 1% false discovery rate was determined by accumulating 1% of reverse database hits. Minimum peptide length was seven amino acids. The first majority protein ID was selected as the representative protein of protein group, and used as protein ID for further analysis. To obtain the quantitative data, label-free quantification (LFQ) with a minimum of two ratio counts was applied. Normalized LFQ intensity was obtained.

### Systemic analysis

Protein localization data was obtained from the Uniprot database and primary localization is used for further analysis. Biological process terms of gene ontology (GO) analysis was obtained using DAVID (https://david.ncifcrf.gov/).

### Fluorescent microscopy

EVs were spread on a microscopy slide (superfrost +) and allowed to attach to the surface overnight at 4 °C. Slides were washed three times with PBS and then blocked for 30 minutes using 1% BSA in PBS. For permeabilization, samples were incubated with 0.1% Tween-20 in PBS for 5 minutes and then washed three times with PBS before the blocking step. After blocking, the samples were incubated with primary antibodies against either STX4, SCAMP3, or beta-actin which were diluted in PBS containing 1% BSA for 1 h at room temperature, washed with PBS, and then incubated with secondary antibodies which were diluted in PBS containing 1% BSA for 30 minutes at room temperature. Another three washes with PBS followed. PKH26 Red Fluorescent Cell Linker Kit (Sigma Aldrich) was used to stain the EV membrane and samples were mounted using DAPI containing mounting medium. Samples were imaged with an Axio Observer (Zeiss). The exposure time for the two channels evaluated was constant for all the samples. Computational analysis was done using the ZEN Blue and ImageJ software.

### Flow cytometry

EV analysis with flow cytometry was performed using antibody coated beads. The anti-CD63 coated beads were commercially acquired (Exosome-Human CD63 isolation/detection reagent (from cell culture media), Thermo Fisher Scientific), while the anti-STX4 and anti-SCAMP3 coated beads were made using a bead conjugation kit (Dynabeads Antibody Coupling Kit, Thermo Fisher Scientific) according to manufacturer’s instructions. Beads were washed with PBS and were then incubated with 15 μg of EVs overnight at 4 °C. After incubation, samples were washed 3 times with PBS containing 1% EV-depleted FBS followed by 15 minutes incubation with human IgG antibody at 4 °C. After another three washes, the samples were incubated for 40 minutes with primary antibodies against either CD63 (PE Mouse Anti-Human CD63, BD Pharmingen), CD81 (PE Mouse Anti-Human CD81, BD Pharmingen), STX4, or SCAMP3 diluted in PBS containing 1% EV-depleted FBS. Samples were washed and incubated for 20 minutes with Alexa 488 conjugated secondary antibodies for STX4 and SCAMP3 after which another wash was performed. Samples were run in a FACSAria (BD Pharmingen) and results were analyzed using the FlowJo software (Tri Star).

## Additional Information

**How to cite this article**: Cvjetkovic, A. *et al*. Detailed Analysis of Protein Topology of Extracellular Vesicles - Evidence of Unconventional Membrane Protein Orientation. *Sci. Rep.* **6**, 36338; doi: 10.1038/srep36338 (2016).

**Publisher’s note:** Springer Nature remains neutral with regard to jurisdictional claims in published maps and institutional affiliations.

## Supplementary Material

Supplementary Dataset 1

Supplementary Dataset 2

Supplementary Dataset 3
